# γ' Fibrinogen as a Predictor of Survival in Amyotrophic Lateral Sclerosis

**DOI:** 10.3389/fcvm.2021.715842

**Published:** 2021-09-09

**Authors:** Ana Catarina Pronto-Laborinho, Catarina S. Lopes, Vasco A. Conceição, Marta Gromicho, Nuno C. Santos, Mamede de Carvalho, Filomena A. Carvalho

**Affiliations:** ^1^Instituto de Medicina Molecular, Faculdade de Medicina, Universidade de Lisboa, Lisbon, Portugal; ^2^Department of Neurosciences and Mental Health, Hospital de Santa Maria, Centro Hospitalar Universitário Lisboa Norte (CHULN), Lisbon, Portugal

**Keywords:** amyotrophic lateral sclerosis, prognosis, respiratory function, survival, gamma' fibrinogen

## Abstract

Amyotrophic lateral sclerosis (ALS) is an aggressive neurodegenerative disorder related to neuroinflammation that is associated with increased risk of thrombosis. We aimed to evaluate γ' fibrinogen plasma level (an *in vivo* variant of fibrinogen) as a biomarker in ALS, and to test its role as a predictor of disease progression and survival. Sixty-seven consecutive patients with ALS were followed and the results were compared with those from 82 healthy blood donors. Patients were clinically evaluated at the time of blood sampling and on follow-up (every 3 months for the beginning of the follow-up until death) by applying the revised ALS Functional Rating Scale. Human plasma γ' fibrinogen concentration was quantified using a specific two-site sandwich kit enzyme-linked immunosorbent assay. We found, for the first time, a positive association between γ' fibrinogen concentration and survival in ALS patients: patients with higher γ' fibrinogen plasma levels survived longer, and this finding was not influenced by confounders such as age, gender, respiratory impairment, or functionality (ALSFRS-R score). Since increased levels have a positive impact on outcome, this novel biomarker should be further investigated in ALS.

**Graphical Abstract:**
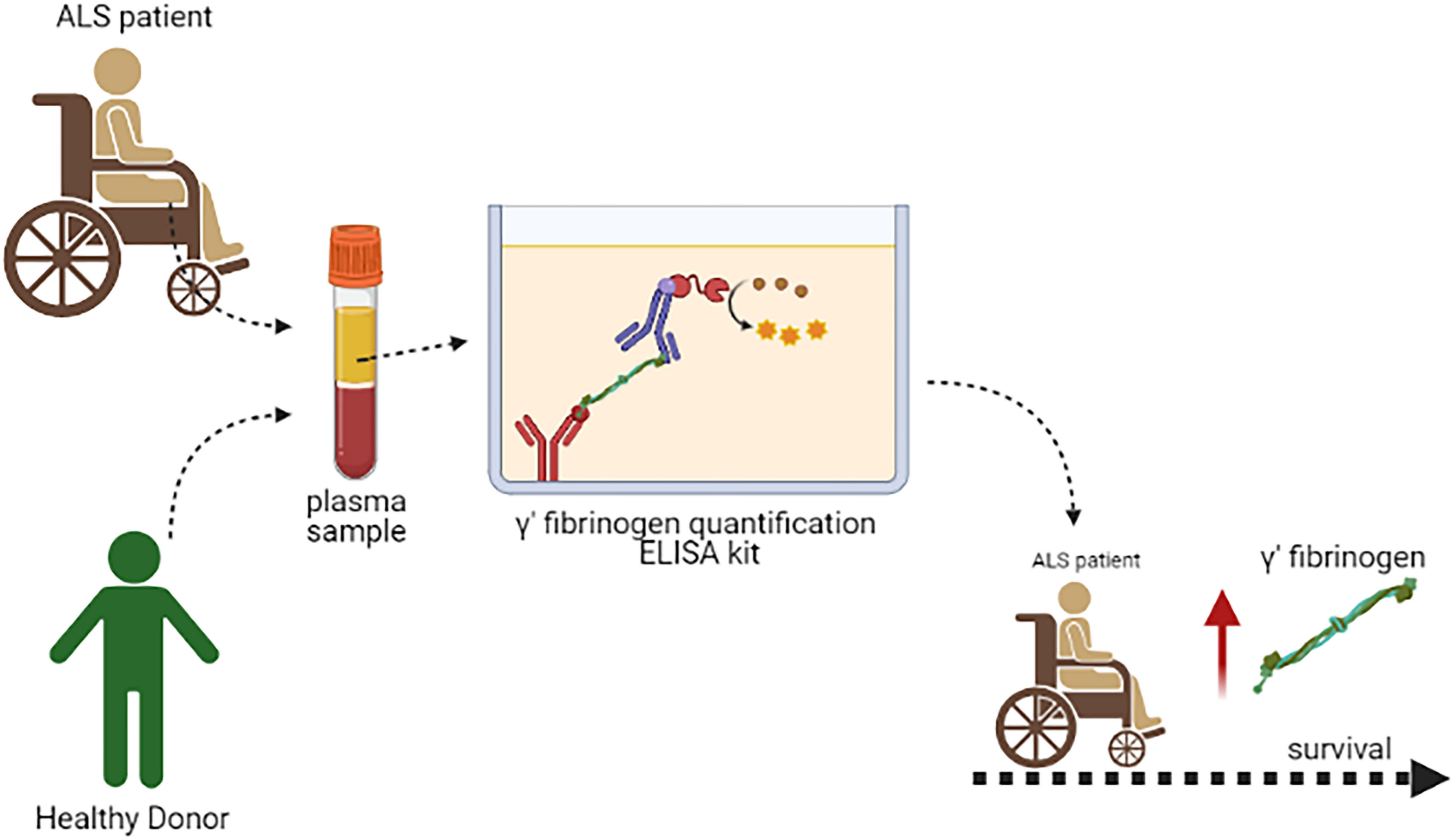
Schematic illustration of the longitudinal assessment of the effects of γ′ fibrinogen on survival in amyotrophic lateral sclerosis (ALS).

## Introduction

Amyotrophic lateral sclerosis (ALS) is a progressive and fatal neurodegenerative disorder, in which there is degeneration of motor neurons in the spinal cord, brainstem, and motor cortex (1). Early diagnosis can be difficult and slow, and there is no disease-modifying therapy available (1). There is therefore a pressing need for novel biomarkers to be used in clinical trials (2). Respiratory dysfunction is the major determinant of functional impairment and death in ALS (3). Hypoxia derives from respiratory muscles weakness, but its role in precipitating further neuronal damage or skeletal muscle dysfunction is unclear (4). Episodes of vascular thrombosis are commonly reported in ALS, in general associated to immobility, but other risk factors have not been deeply explored (5).

There is large evidence that ALS is associated with markers of systemic inflammation (6), possibly increased by respiratory distress (7). The interplay between inflammation and coagulation is an important subject that involves fibrinogen (8). A pro-inflammatory role for fibrinogen has been investigated in several neurological conditions, in particular in spinal cord and brain trauma, stroke, myopathies, multiple sclerosis and Alzheimer's disease (8). Increased fibrinogen levels are associated with higher cardiovascular and venous thromboembolic risk (9).

Gamma' (γ') fibrinogen, a fibrinogen γ chain variant produced *via* alternative mRNA splicing, usually constitutes ~8% of plasma fibrinogen and is elevated in individuals with pathological conditions, such as essential arterial hypertension and venous thromboembolism. Like the most common variant of fibrinogen, γ' fibrinogen has also been associated with inflammation in several studies, and this isoform produces clots that are more resistant to lysis (10). In inflammatory conditions, γ' fibrinogen has its relative percentage increased in the total plasma fibrinogen and is closely correlated with other markers of inflammation (10).

We aimed to investigate γ' fibrinogen as a molecular diagnostic and prognostic factor for ALS as, to the best of our knowledge, this subject was not addressed before.

## Methods

### Subjects

Sixty-seven consecutive patients with ALS followed in our unit were included in this study. Inclusion criteria were probable or definitive disease according to the revised El Escorial criteria (11), with neurophysiological changes supporting diagnosis (12), informed consent, and riluzole medication over, at least, 1 month. Exclusion criteria were pregnancy, smoking, other medical conditions (e.g., hematological diseases, diabetes, lung disorders, heart diseases, previous stroke, or peripheral neuropathy), severe dementia precluding appropriate informed consent, and marked symptoms of respiratory insufficiency, to avoid patient discomfort. No individual in the patient group or in the control group had any reported co-morbidity. The mean body mass index (BMI) of the ALS patients was calculated, as well as the mean BMI among men and women. During the same period, we assessed 82 healthy controls, who were either workers of the academic center or spouses of the patients. This study was approved by the Ethics Committee of Centro Académico de Medicina de Lisboa.

Patients were clinically evaluated at the time of blood sampling and on follow-up (every 3 months for the time of follow-up until death) by applying the functional scale revised—ALS Functional Rating Scale (13), considering its total score and the respiratory subscore (score from questions 10, 11, and 12), and through the assessment of the predicted percentage of forced vital capacity (%FVC). From this group, 78% of ALS cases are called “sporadic,” meaning the cause or causes of the disease are unknown. Nine percent of ALS cases are due to genetic mutations and are inherited from a family member (familial cases).

Furthermore, age, gender, diagnostic delay, region of onset, and presence (or absence) of cognitive changes (by clinical evaluation) were considered, since those variables have been reported as prognostic factors in ALS (14).

### Blood Collection

Human venous blood samples from ALS patients and controls were collected into K_3_EDTA tubes, at the same period of the day (between 8 and 10 a.m.), and immediately centrifuged at 1,040 × *g* for 10 min, as previously described (9). Plasma was collected and stored at −80°C until analyzed.

### γ' Fibrinogen Quantification in Plasma

Human plasma γ' fibrinogen concentration was quantified using a specific two-site sandwich kit enzyme-linked immunosorbent assay (ELISA; Gamma Couer ELISA kit, Gamma Therapeutics, Portland, OR, USA) as used by other authors (9, 15). The γ' fibrinogen assay was performed using human venous plasma samples isolated from blood collected into K_3_EDTA anticoagulant tubes. A monoclonal anti-γ' fibrinogen antibody, specific for the unique carboxyl terminal sequence of the γ' fibrinogen chain, was used as the capture antibody. After incubation with the antibody, unbound non-γ' fibrinogen, such as γAγA fibrinogen, and others plasma components were removed by sequential washing. Secondly, an enzyme-labeled polyclonal anti-fibrinogen detection antibody was added, which binds to the captured γ' fibrinogen, forming a detection antibody sandwich. After this, several sequential washings removed excess detection antibody and a substrate for the horse radish peroxidase (HRP)-labeled detector was added to generate a colored product. Each sample (from either ALS patients or controls) was run in duplicate, and the mean γ' fibrinogen concentration was calculated. Absorbance of the end-product was read at 450 nm on an Infinite M200 microplate reader (Tecan, Männedorf, Switzerland). The γ' fibrinogen concentration was determined using a standard calibration curve prepared with solutions of different concentrations of this protein. The coefficient of variation (CV) between kits was quantified. The intra-assay CV is calculated as the average of the CVs of all samples in a given assay or set of assays. The inter-assay CV is calculated from the mean of the means ± standard deviation (SD) of a low and high γ' fibrinogen plasma control sample included in each kit. Intra- and inter-assay CVs below 10% are considered as good. Inter-assays values for the high control γ' fibrinogen concentration results in a value of 45.86 ± 0.61 mg/dL (mean of means ± SD) with a CV = 1.32% and in 32.54 ± 1.53 mg/dL (mean of means ± SD), CV = 4.70% for the low concentration control γ' fibrinogen in plasma. The intra-assays CV for healthy donors (*n* = 82) was 5.05 ± 3.72%, and for ALS patients (*n* = 67) it was 4.67 ± 3.04%.

### Determination of the Predicted Forced Vital Capacity

Forced vital capacity (FVC) was determined with the patients in sitting position, using a computer-based USB spirometer (microQuark, Cosmed, Chicago, IL, USA). The best of three consistent tests was used to define the %FVC.

### Statistical Analysis

Three types of statistical analyses were done for the obtained clinical and experimental data: (1) between-group comparisons to test whether γ' fibrinogen values differed between groups (ALS patients vs. controls) were compared using the parametric Student's *t*-test and the non-parametric Mann-Whitney *U*-test, and whether those two groups were well-matched demographically; (2) baseline assessments of the relation between γ' fibrinogen and either ALS diagnosis or the values of the clinical variables of interest (ALSFRS-R global and respiratory scores and %FVC) controlling for possible demographic and clinical confounders; and, (3) longitudinal assessments of the relation between γ' fibrinogen and ALS progression. Specifically, for (2) we performed separate multiple regressions on patients' ALSFRS-R and %FVC values (dependent variables), considering as independent variables γ' fibrinogen level, age, gender, presence of comorbid frontotemporal dementia (FTD), region of onset (spinal vs. bulbar vs. respiratory) and diagnosis delay. For (3) we underwent two approaches: firstly, we performed multiple regressions on the change of ALSFRS-R scores and %FVC values per year (dependent variables) that controlled for the aforementioned independent variables (14); secondly, we performed Cox regressions that controlled for the same independent variables, by categorizing ALS patients according to γ' fibrinogen levels (reference group, group 1: <30 mg/dL; group 2: 30–60 mg/dL; group 3: >60 mg/dL), and using continuous γ' fibrinogen levels. In both Cox regressions, the endpoint was set at 5 years after diagnosis (censoring point). Separate coefficients were used for spinal- and bulbar-onset patients in all regressions, as detailed above. Bonferroni correction was applied to the baseline and longitudinal multiple regressions (significant value: *p* < 0.05/3).

## Results

### Sample Characteristics and Between-Group Comparisons

**Figure 1:**
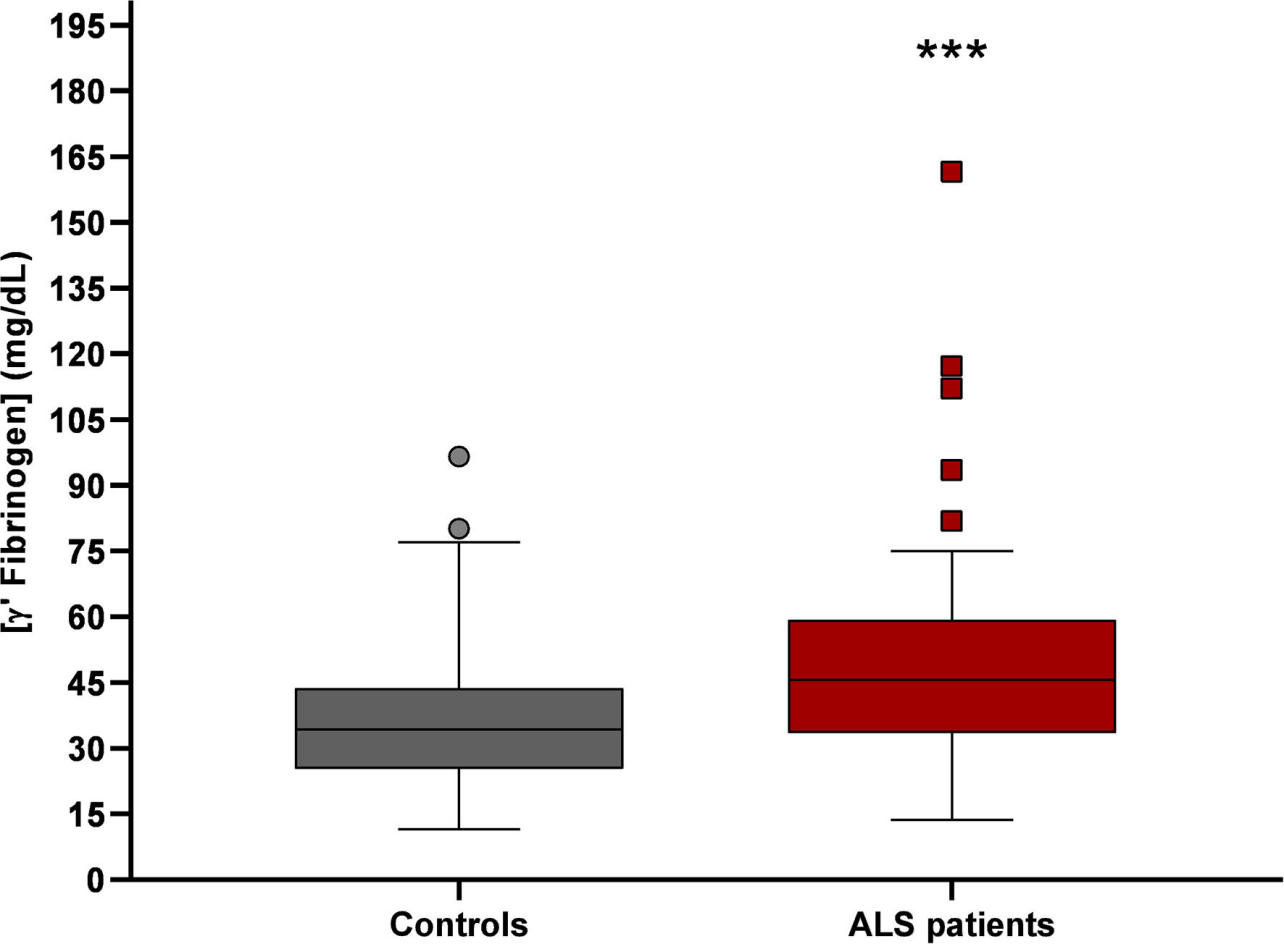
γ' fibrinogen levels in plasma from controls (*n* = 82) and ALS patients (*n* = 67). γ' fibrinogen values of ALS patients were significantly higher than in controls (***p = 0.0006).

The sample of ALS patients consisted of 40 men (59.7%) and 27 women (40.3%), with a mean age of 63.3 ± 12.3 years (mean ± SD) and a mean disease duration of 24.4 ± 21.4 months (mean ± SD). The control group consisted of 46 men (56.1%) and 36 women (43.9%), with a mean age of 47.3 ± 11.6 years (mean ± SD). Gender distribution was similar across groups (*p* > 0.30), but controls were significantly younger (*p* < 0.001; Supplementary Table 1). No statistically significant differences were observed between BMI of healthy donors' (25.91 ± 3.85 kg/m^2^, mean ± SD) and ALS patients' (25.06 ± 2.74 kg/m^2^, mean ± SD; *p* = 0.27; Supplementary Table 1). γ' fibrinogen levels were significantly higher in ALS patients [51.6 ± 24.5 mg/dL, mean ± SD, lower 95% confidence interval (CI) = 45.6; upper 95% confidence interval (CI) = 57.6) than in controls (38.7 ± 16.6 mg/dL; mean ± SD, lower 95% confidence interval (CI) = 35.0; upper 95% confidence interval (CI) = 42.3); *p* = 0.0006; Figure 1].

### Baseline Assessment of the Relation Between γ' Fibrinogen and Either the Diagnosis of, or Global or Respiratory Function in, ALS

Consistently with the results from the direct comparison of γ' fibrinogen values between patients and controls, multiple regression analysis showed that γ' fibrinogen concentration was increased in ALS patients when controlling for age and gender (*p* = 0.017; Supplementary Table 2). In ALS, γ' fibrinogen levels were not influenced by gender, age, diagnosis delay, region of onset, or clinical signs of dementia (all *p* > 0.25). Additionally, we did not detect any association between γ' fibrinogen levels and the clinical variables of interest (ALSFRS-R global score, ALSFRS-respiratory subscore, and %FVC; all *p* > 0.25; Supplementary Table 3).

### Longitudinal Assessment of the Relation Between γ' Fibrinogen and Either Global or Respiratory Function in ALS

Regression analysis disclosed that ALSFRS-R was positively associated with γ' fibrinogen levels, but it was not significant after Bonferroni correction (β${}_{{\text{γ}}^{\prime }fibrinogen}$ = 0.228; *p* = 0.034). There was no association with ALSFRS-R-respiratory subscore or FVC (*p* > 0.1).

### Longitudinal Assessment of the Effects of γ' Fibrinogen on Survival in ALS

**Figure 2:**
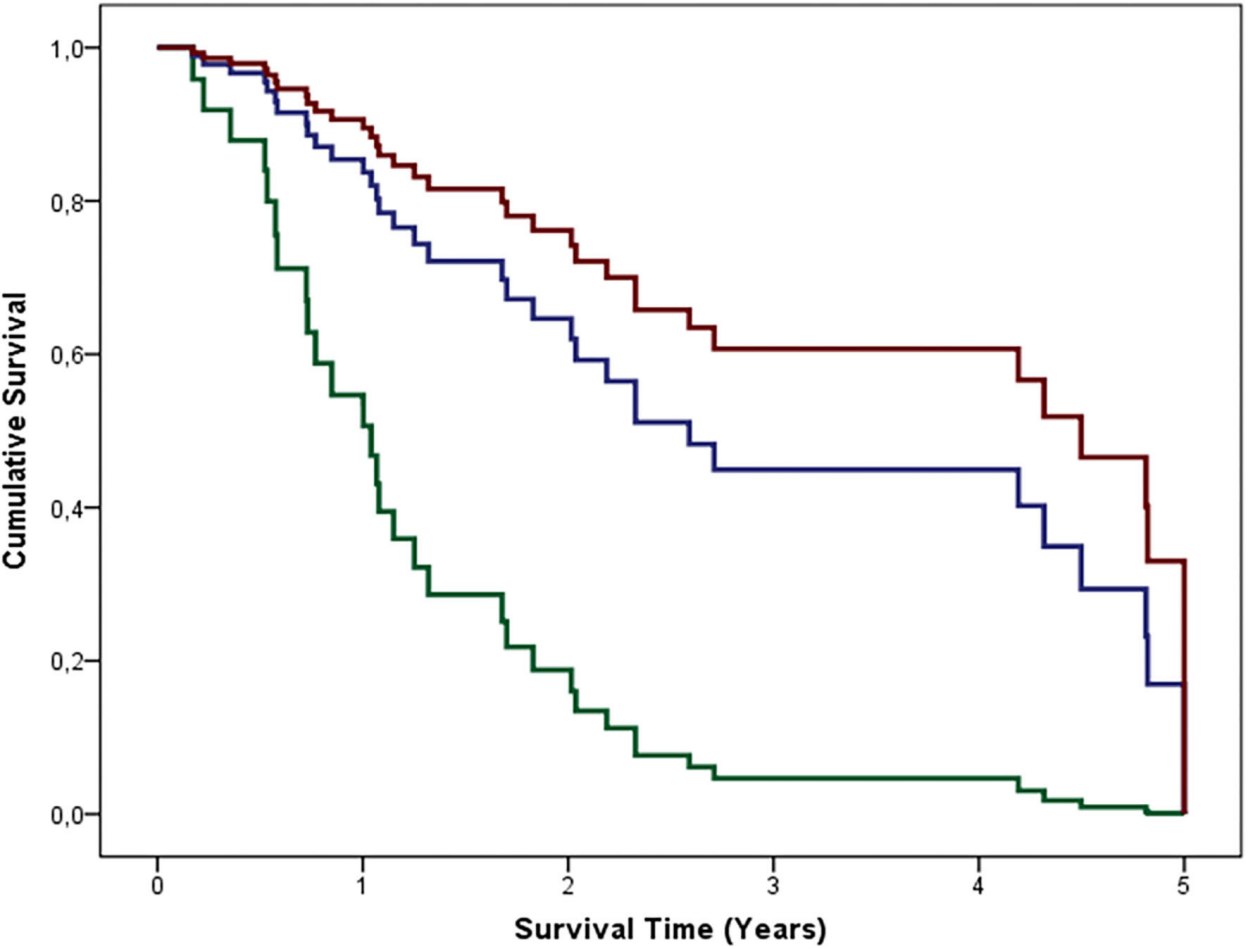
Cumulative survival of 50 ALS patients grouped according to their γ' values. Group 1 or reference group (green line): γ' fibrinogen <30 mg/dL; group 2 (blue line): γ' fibrinogen 30–60 mg/dL; group 3 (red line): γ' fibrinogen > 60 mg/dL. The depicted curves are based on the mean of the independent variables (gender, age, FTD, spinal and/or bulbar onset, diagnosis delay, and the aforementioned γ' fibrinogen-related groups; Supplementary Table 5A).

The survival analysis (Cox proportional hazard regressions) provided strong evidence toward an independent association between higher γ' fibrinogen levels and longer survival (Figure 2; Supplementary Table 5). Both the concentrations of γ' fibrinogen between 30 and 60 mg/dL (group 2; *B* = −1.343; *p* = 0.011) and those above 60 mg/dL (group 3; *B* = −1.814; *p* = 0.003) were associated with longer survival, when compared to γ' fibrinogen concentrations below 30 mg/dL (group 1; reference group; Supplementary Table 5). Moreover, when coding γ' fibrinogen values continuously, higher γ' fibrinogen values was an independent predictor of longer survival (*B* = −0.014; *p* = 0.045; Supplementary Table 5). Survival analysis showed, as expected, a negative association between age and survival (*B* = 0.039; *p* = 0.036 for γ' fibrinogen values coded categorically; *B* = 0.040; *p* = 0.029 for γ' fibrinogen values coded continuously). The average survival in our population was similar to other ALS studies (14).

## Discussion

We aimed to investigate γ' fibrinogen as an inflammatory biomarker in ALS. The plasma concentration of this fibrinogen variant was higher in ALS patients than in controls, which did not seem to depend on gender nor age. The levels identified in controls are similar to the values published elsewhere (16). We found, to our best knowledge for the first time, a link between γ' fibrinogen concentration and survival in ALS patients: patients with higher γ' fibrinogen plasma levels survived longer, and this finding was not influenced by confounders like age, gender, respiratory impairment, or functionality (ALSFRS-R score). Moreover, these results were quite robust, as they were obtained both when categorizing patients in three groups, according to γ' fibrinogen levels, and when considering continuous titers for γ' fibrinogen levels.

Several studies have reported that total fibrinogen levels vary with age and gender. However, from the available literature, with several divergent opinions, the association between γA/γ' fibrinogen levels either with age or gender is not clear. It is not known if there is a direct and independent association of age with the increase of fibrinogen values. Lovely et al. found that the mean γA/γ' fibrinogen levels were slightly higher in females than in males, but without statistical significance (*p* = 0.207), in healthy blood donors (16). This result is in contrast to the levels of total fibrinogen in healthy females' plasma, which have been shown to be significantly higher than in healthy males. These results indicate that γA/γ' fibrinogen levels are independent of gender and age, even when total fibrinogen levels are gender dependent (16).

Fibrinogen is increased in thromboembolic phenomena and inflammation. In the central nervous system, fibrinogen can induce signaling networks *via* binding sites for multiple receptors and proteins, acting as a mediator of neurodegeneration (17). The pro-inflammatory functions of fibrinogen are associated with signaling through binding sites that are not overlapping with those involved in the coagulation cascade (18). In particular, integrin receptor activation triggers a pro-inflammatory effect by activating a cascade of events that involves microglia activation (17, 18). In ALS, there is a systemic low-grade inflammation (19), with increased levels of plasma fibrinogen, high erythrocyte sedimentation rate, C-reactive protein, and neutrophil to lymphocyte ratio. Interleukin-6 was found to be elevated in ALS, in particular in patients with diaphragm weakness (7).

Remarkably, we found that increased levels of γ' fibrinogen can have a neuroprotective role, increasing survival of ALS patients, which is against a possible action promoting neuro-inflammation and causing neuronal degeneration. Since fibrinogen activates microglia (18, 19) in the central nervous system, we speculate that a specific activity in stimulating the alternative activation (M2) could promote a protective outcome (20).

Nevertheless, our study has a number of limitations. Namely, we did not measure total fibrinogen levels or other markers of inflammation, and we did not quantify γ' fibrinogen longitudinally. However, the large number of patients and controls and the robustness of our findings regarding the association between γ' fibrinogen and survival suggests that this new avenue should be further investigated. The potential neuroprotective role of γ' fibrinogen, moreover, should lead to additional research in other neurodegenerative disorders.

## Data Availability Statement

The original contributions presented in the study are included in the article/Supplementary Material, further inquiries can be directed to the corresponding author/s.

## Ethics Statement

The studies involving human participants were reviewed and approved by the Ethics Committee of Centro Académico de Medicina de Lisboa. The patients/participants provided their written informed consent to participate in this study. The study is conformed to the standards defined in the latest revision of the Declaration of Helsinki.

## Author Contributions

ACP-L and CSL: acquisition and analysis of the data. VAC: statistical analysis. MG: collection of ALS patients' clinical data. MC: clinical selection of ALS patients. FAC and NCS: study concept and design. All authors: drafting the manuscript and/or figures.

## Funding

This work was supported by UID/BIM/50005/2019 and PTDC/EMD-TLM/7289/2020, a project funded by Fundação para a Ciência e a Tecnologia - Ministério da Ciência, Tecnologia e Ensino Superior (FCT-MCTES), through Fundos do Orçamento de Estado (Portugal). CSL also acknowledges FCT-MCTES fellowship PD/BD/135045/2017.

## Conflict of Interest

The authors declare that the research was conducted in the absence of any commercial or financial relationships that could be construed as a potential conflict of interest.

## Publisher's Note

All claims expressed in this article are solely those of the authors and do not necessarily represent those of their affiliated organizations, or those of the publisher, the editors and the reviewers. Any product that may be evaluated in this article, or claim that may be made by its manufacturer, is not guaranteed or endorsed by the publisher.
