# Corrigendum: γ′ Fibrinogen as a Predictor of Survival in Amyotrophic Lateral Sclerosis

**DOI:** 10.3389/fcvm.2021.800151

**Published:** 2021-11-15

**Authors:** Ana Catarina Pronto-Laborinho, Catarina S. Lopes, Vasco A. Conceição, Marta Gromicho, Nuno C. Santos, Mamede de Carvalho, Filomena A. Carvalho

**Affiliations:** ^1^Instituto de Medicina Molecular, Faculdade de Medicina, Universidade de Lisboa, Lisbon, Portugal; ^2^Department of Neurosciences and Mental Health, Hospital de Santa Maria, Centro Hospitalar Universitário Lisboa Norte (CHULN), Lisbon, Portugal

**Keywords:** amyotrophic lateral sclerosis, prognosis, respiratory function, survival, gamma′ fibrinogen

The Funding statement had an error in the reference of one project. Instead of PTDC/EMD-TLM/4644/2021, it should be PTDC/EMD-TLM/7289/2020.

## Funding

This work was supported by UID/BIM/50005/2019 and PTDC/EMD-TLM/7289/2020, a project funded by Fundação para a Ciência e a Tecnologia - Ministério da Ciência, Tecnologia e Ensino Superior (FCT-MCTES), through Fundos do Orçamento de Estado (Portugal). CSL also acknowledges FCT-MCTES fellowship PD/BD/135045/2017.

The authors apologize for this error and state that this does not change the scientific conclusions of the article in any way. The original article has been updated.

## Publisher's Note

All claims expressed in this article are solely those of the authors and do not necessarily represent those of their affiliated organizations, or those of the publisher, the editors and the reviewers. Any product that may be evaluated in this article, or claim that may be made by its manufacturer, is not guaranteed or endorsed by the publisher.

