# Supplementary material for: γ' Fibrinogen as a Predictor of Survival in Amyotrophic Lateral Sclerosis
Source: Front Cardiovasc Med. 2021 Sep 9;8:715842. doi: 10.3389/fcvm.2021.715842 (PMC8458885; doi:10.3389/fcvm.2021.715842)
Supplement: Supplementary file 1 [file Data_Sheet_1.docx]

γ’ fibrinogen as a predictor of survival in amyotrophic lateral sclerosis

Ana Catarina Pronto-Laborinho ^1†^, Catarina S. Lopes^1†^, Vasco A. Conceição^1^, Marta Gromicho^1^, Nuno C. Santos^1^, Mamede de Carvalho^1,2^, Filomena A. Carvalho^1*^

Supplementary Material

**Table S1**| Sample characteristics and baseline comparisons of demographic variables between patients with ALS and controls. Continuous variables (such as age and γ’ fibrinogen plasma concentration) were compared between groups using parametric (two-sample *t*-) or non-parametric (Mann-Whitney U) tests, depending on whether data normality was not or was rejected. Data normality was assessed through Lilliefors tests. Gender prevalence was compared across groups through a $\chi^{2}$-test. Sample sizes available for each demographic or clinical variable are indicated by *n*_Controls_ and *n*_ALS_ for controls and patients with ALS, respectively. Values are in percentage (%) or mean ± standard deviation (SD).

|  |  | | ***Control group*** | ***ALS patients’ group*** | $\boldsymbol{p}$***-value*** |
| --- | --- | --- | --- | --- | --- |
| ***Demographic data*** *(inclusion criteria)* | | | | | |
| ***n*_Controls_ = 82**  ***n*_ALS_ = 67** | **Age at diagnosis (years)** | | 47.3 ± 11.6 | 63.3 ± 12.3 | ***p < 0.001*** |
|  | **Gender** | **male** | 56.1% | 59.7% | *p > 0.30* |
|  |  | **female** | 43.9% | 40.3% |  |
|  | **BMI (kg/m^2^)** | | 25.91 ± 3.81 | 25.06 ± 2.74 | *p = 0.27* |
| ***Clinical data*** *(inclusion criteria)* | | | | | |
| ***n*_ALS_ = 67** | **Onset form** | | N.A. | Spinal: 61.2%  Bulbar: 34.3%  Respiratory: 4.5% |  |
|  | **Fronto-temporal dementia (FTD)** | | N.A. | 3.0% |  |
| ***n*_ALS_ = 47** | **ALSFRS-R**  **Global scores** | | N.A. | 30.52±6.66 |  |
|  | **ALSFRS-R Respiratory (sub)scores** | | N.A. | 10.58±2.00 |  |
|  | **%FVC** | | N.A. | 83.47±18.96 |  |
| Abbreviations: ALS, amyotrophic lateral sclerosis; BMI, body mass index; FTD, fronto-temporal dementia; ALSFRS-R, amyotrophic lateral sclerosis functional scale - revised; %FVC, predicted forced vital capacity; SD, standard deviation; N.A., non-applicable. | | | | | |

**Table S2** | Results of the multiple-regression analysis between γ’ fibrinogen values (at the beginning of the study), a diagnosis of ALS, gender, and age. The effects of the independent variables on γ’ fibrinogen values are expressed by the corresponding regression coefficients (*β* values). Significant and positive coefficients mean that higher values of the independent variables are associated with higher γ’ fibrinogen values; *n* denotes the sample size. Significant coefficients are shown in bold.

| **γ’ Fibrinogen**  ***n* = 141** | **Independent Variable** | ***𝛃*** | ***p*** |
| --- | --- | --- | --- |
|  | *Intercept* | **28.956** | < 0.0001 |
|  | ALS | **9.967** | 0.017 |
|  | Gender | 4.567 | 0.159 |
|  | Age | 0.163 | 0.256 |
| Abbreviations: ALS, amyotrophic lateral sclerosis. | | | |

**Table S3** | Results of the baseline assessment of the relation between γ’ fibrinogen plasma concentration and either global or respiratory function in ALS. ALS Functional Rating Scale – Revised (ALSFRS-R) global and respiratory, or R, scores were all assessed, like the predicted forced vital capacity (%FVC), at diagnosis. Such initial assessment (or baseline) values were used as the dependent variables in the multiple regressions shown here, which controlled for γ' fibrinogen levels, as well as for gender (0 and 1 for male and female participants, respectively), age, comorbid frontotemporal dementia (FTD), onset form (with spinal and bulbar onset forms being coded in a binary fashion, to be compared with respiratory onset form), and diagnosis delay (see main text for rationale). The effects of the independent variables on the dependent variables [a) ALSFRS-R global score; b) ALSFRS-R R score; c) %FVC] are expressed by the corresponding regression coefficients (*β*), with significant, positive (negative, resp.) coefficients meaning that higher values of the independent variables are associated with higher (lower, resp.) values of dependent variable. In all regressions, *n* denotes the sample size. *β*_0_ denotes the intercept. Coefficients with *p*-values below 0.05, 0.01, and 0.001 are indicated with *, **, and ***, respectively. Coefficients with *p*-values below 0.05/3 (*i.e.*, significant post-Bonferroni correction) are also shown in bold.

| **Regression coefficient**  **Dependent variable** | $\boldsymbol{\beta}_{\boldsymbol{0}}$ | $\boldsymbol{\beta}_{\mathbf{gender}}$ | $\boldsymbol{\beta}_{\mathbf{age}}$ | $\boldsymbol{\beta}_{\mathbf{FTD}}$ | $\boldsymbol{\beta}_{\mathbf{spinal onset}}$ | $\boldsymbol{\beta}_{\mathbf{bulbar onset}}$ | $\boldsymbol{\beta}_{\mathbf{diagnosis delay}}$ | $\boldsymbol{\beta}_{\mathbf{fibrinogen}}$ |
| --- | --- | --- | --- | --- | --- | --- | --- | --- |
| **(a) ALSFRS-R**  **Global score**  **(*n* = 56)** | **52.842***** | -0.061 | -0.170 | 4.855 | -1.735 | -0.961 | -1.059 | -0.008 |
| ***p*-value** | < 10^-7^ | 0.978 | 0.083 | 0.363 | 0.667 | 0.826 | 0.060 | 0.831 |
| **(b) ALSFRS-R**  **R score**  **(*n* = 56)** | **11.718***** | 1.268* | -0.022 | 0.258 | 0.825 | -0.759 | -0.056 | -0.005 |
| ***p-*value** | < 10^-6^ | 0.022 | 0.361 | 0.844 | 0.406 | 0.480 | 0.682 | 0.598 |
| **(c) %FVC**  **(*n* = 48)** | **111.270***** | -6.968 | -0.599* | 8.336 | 6.586 | 12.600 | 0.065 | 0.097 |
| ***p-*value** | < 10^-4^ | 0.297 | 0.042 | 0.678 | 0.636 | 0.397 | 0.968 | 0.392 |
| Abbreviations: ALS, amyotrophic lateral sclerosis; FTD, fronto-temporal dementia; ALSFRS-R, amyotrophic lateral sclerosis functional scale revised; % FVC, predicted forced vital capacity. | | | | | | | | |

**Table S4 |** Results of the longitudinal assessment of the relation between γ’ fibrinogen plasma concentration levels and either global or respiratory function in ALS. ALS Functional Rating Scale – Revised (ALSFRS-R) global and respiratory, or R, scores were assessed, like the predicted forced vital capacity (%FVC), in intervals of about 3-6 months. The yearly change in such variables ($\Delta$ALSFRS-R global score, $\Delta$ALSFRS-R R score, and $\Delta$%FVC) was calculated using the respective first and third measurements; such yearly change values were used as the dependent variables in the multiple regressions shown here, which controlled for γ' fibrinogen levels, as well as for gender (0 and 1 for male and female participants, respectively), age, comorbid frontotemporal dementia (FTD), onset form (with spinal and bulbar onset forms being coded in a binary fashion, to be compared with respiratory onset form), and diagnosis delay (see main text for rationale). The effects of the independent variables on the dependent variables [a) $\Delta$ALSFRS-R global score; b) $\Delta$ALSFRS-R R score; c) $\Delta$%FVC] are expressed by the corresponding regression coefficients (*β*), with significant, positive (negative, resp.) coefficients meaning that higher values of the independent variables are associated with higher (lower, resp.) values of dependent variable. All patients included in the %FVC multiple regression had spinal onset; therefore, no spinal- or bulbar-onset coefficients were included in such regression [hence, the non-applicable (N.A.)]. In all regressions, *n* denotes the sample size. *β*_0_ denotes the intercept. Coefficients with *p*-values below 0.05, 0.01, and 0.001 are indicated with *, **, and ***, respectively. Coefficients with *p*-values below 0.05/3 (*i.e.*, significant post-Bonferroni correction) are also shown in bold.

| **Regression coefficient**  **Dependent variable** | $\boldsymbol{\beta}_{\boldsymbol{0}}$ | $\boldsymbol{\beta}_{\mathbf{gender}}$ | $\boldsymbol{\beta}_{\mathbf{age}}$ | $\boldsymbol{\beta}_{\mathbf{FTD}}$ | $\boldsymbol{\beta}_{\mathbf{spinal onset}}$ | $\boldsymbol{\beta}_{\mathbf{bulbar onset}}$ | $\boldsymbol{\beta}_{\mathbf{diagnosis delay}}$ | $\boldsymbol{\beta}_{\boldsymbol{\gamma}^{\mathbf{'}}\mathbf{fibrinogen}}$ |
| --- | --- | --- | --- | --- | --- | --- | --- | --- |
| **(a)** $\boldsymbol{\Delta}$**ALSFRS-R**  **Global score**  **(*n* = 46)** | 2.317 | -6.064 | -0.335 | 4.238 | -5.984 | -3.327 | 1.829 | 0.228* |
| ***p*-value** | 0.878 | 0.168 | 0.070 | 0.655 | 0.485 | 0.717 | 0.076 | 0.034 |
| **(b)** $\boldsymbol{\Delta}$**ALSFRS-R**  **R score**  **(*n* = 46)** | -2.299 | -0.052 | -0.021 | 1.471 | -3.157 | -3.232 | 0.436 | 0.060 |
| ***p-*value** | 0.672 | 0.973 | 0.741 | 0.666 | 0.307 | 0.331 | 0.233 | 0.114 |
| **(c)** $\boldsymbol{\Delta}$**%FVC**  **(*n* = 12)** | 0.100 | -2.312 | -0.264 | -11.147 | N.A. | N.A. | 2.241* | 0.096 |
| ***p-*value** | 0.994 | 0.661 | 0.216 | 0.196 | N.A. | N.A. | 0.021 | 0.172 |
| Abbreviations: ALS, amyotrophic lateral sclerosis; FTD, fronto-temporal dementia; ALSFRS-R, amyotrophic lateral sclerosis functional scale - revised; %FVC, predicted forced vital capacity; N.A., non-applicable. | | | | | | | | |

**Table S5** | Results of the Cox proportional hazard regressions evaluating how survival was modulated by γ' fibrinogen values [a) coded categorically: group 1 (γ' fibrinogen concentration below 30 mg/dL); group 2 (concentration between 30 and 60 mg/dL); group 3 (concentration above 60 mg/dL; b) coded continuously], as well as by demographic and clinical variables. Positive (*B* > 0) and negative (*B* < 0) coefficients denote an association between a given predictor and lower and higher survival, respectively (Figure 2). Gender was coded with 0 and 1, for male and female participants, respectively. 50 patients were included in the regressions. Significant coefficients are shown in bold.

|  | **Independent Variables** | ***B*** | **Exp (B)** | ***p*-value** |
| --- | --- | --- | --- | --- |
| **a)**  **γ' fibrinogen values categorized into groups** | Gender | -0.431 | 0.650 | 0.265 |
|  | Age | **0.039** | 1.039 | 0.036 |
|  | FTD | 1.997 | 7.369 | 0.092 |
|  | Spinal onset | -1.471 | 0.230 | 0.183 |
|  | Bulbar onset | 0.351 | 1.420 | 0.745 |
|  | Diagnosis delay | -0.011 | 0.989 | 0.925 |
|  | Group 2 (γ' fibrinogen 30–60 mg/dL) | **-1.343** | 0.261 | 0.011 |
|  | Group 3 (γ' fibrinogen > 60 mg/dL) | **-1.814** | 0.163 | 0.003 |
| **b)**  **γ' fibrinogen continuous values** | Gender | -0.591 | 0.554 | 0.112 |
|  | Age | **0.040** | 1.040 | 0.029 |
|  | FTD | 1.510 | 4.528 | 0.183 |
|  | Spinal onset | -1.037 | 0.355 | 0.333 |
|  | Bulbar onset | 0.451 | 1.569 | 0.675 |
|  | Diagnosis delay | -0.040 | 0.961 | 0.709 |
|  | γ' fibrinogen levels | **-0.014** | 0.986 | 0.045 |
| Abbreviations: FTD, fronto-temporal dementia. | | | | |
